# The Role of Rotational Thromboelastometry in High-Risk-of-Bleeding Endoscopic Procedures in Patients with Decompensated Liver Cirrhosis

**DOI:** 10.3390/diagnostics16091289

**Published:** 2026-04-25

**Authors:** Irina Gîrleanu, Laura Huiban, Cristina Muzica, Camelia Cojocariu, Cătălin Victor Sfarti, Stefan Chiriac, Sebastian Zenovia, Gheorghe G. Balan, Raluca Avram, Ana Maria Sîngeap, Iulian Buzincu, Ana Maria Trofin, Ioana-Miruna Balmuș, Carol Stanciu, Anca Trifan

**Affiliations:** 1 Faculty of Medicine, “Grigore T. Popa” University of Medicine and Pharmacy, 700115 Iasi, Romania; gilda_iri25@yahoo.com (I.G.); anamaria.singeap@yahoo.com (A.M.S.); balmus.ioanamiruna@yahoo.com (I.-M.B.);; 2 Institute of Gastroenterology and Hepatology, “St. Spiridon” County Emergency Clinical Hospital, 700111 Iasi, Romania; 3 Anesthesia and Intensive Care, “St. Spiridon” County Emergency Clinical Hospital, 700111 Iasi, Romania; 4 General Surgery and Liver Transplant Clinic, “St. Spiridon” County Emergency Clinical Hospital, 700111 Iasi, Romania; 5 CENEMED Platform for Interdisciplinary Research, 700115 Iasi, Romania; 6 Department of Exact Sciences and Natural Sciences, Institute of Interdisciplinary Research, “Alexandru Ioan Cuza” University of Iasi, Alexandru Lapusneanu Street, No. 26, 700057 Iasi, Romania

**Keywords:** ROTEM, endoscopic procedures, liver cirrhosis, bleeding, blood product transfusion, coagulopathy

## Abstract

**Background/Objectives**: This study aimed to evaluate the differences between two blood product transfusion protocols [a standard coagulation (SC) group and a rotational thromboelastometry (ROTEM) group] in patients with decompensated liver cirrhosis (LC) undergoing high-bleeding-risk endoscopic procedures. **Methods**: Between December 2024 and March 2025, we conducted a prospective cohort study including adult decompensated cirrhotic patients who needed prophylactic blood product transfusion before high-bleeding-risk endoscopic procedures. The prophylactic blood product transfusion strategy in the SC group was based on conventional coagulation tests (INR, platelets, and fibrinogen), and in the ROTEM group on viscoelastic parameters. **Results**: A total of 72 patients were included in this study (36 patients in each group); most were male (63.9%), Child–Pugh B (54.2%), and had LC with a predominance of alcoholic etiology (51.4%). There were no clinically significant differences regarding the baseline characteristics between the study groups. The most frequent endoscopic procedure was polypectomy (76.4%). Postinterventional bleeding occurred after eight procedures in the SC group and after four procedures in the ROTEM group (*p* = 0.206). Endoscopic hemostasis was effective. Patients from the ROTEM group received fewer FFP transfusions than the SC group (5.6% vs. 69.4%; *p* < 0.0001). Blood product transfusion was needed less in patients evaluated using ROTEM compared with the SC group (41.2% vs. 100%; *p* < 0.0001). There were no differences in the length of hospital stay (*p* = 0.618) or 30-day mortality (*p* = 0.643) between the two study groups. **Conclusions**: ROTEM-guided transfusion management was associated with a significant reduction in blood product use compared with standard coagulation test-based management. However, this difference should be interpreted in the context of the distinct transfusion thresholds applied in the two groups, as the standard coagulation arm followed predefined laboratory-based criteria that may have increased the likelihood of prophylactic transfusion. No statistically significant differences were observed in bleeding complications, length of hospital stay, or 30-day mortality. Therefore, these findings reflect differences in transfusion strategies rather than demonstrating clinical superiority of ROTEM-based management and should be considered preliminary.

## 1. Introduction

Liver cirrhosis (LC) is a progressive and chronic disorder characterized by extensive fibrosis and the formation of regenerative nodules, which disrupt the normal architecture and physiological functions of the liver [1]. This pathological remodeling often results in significant alterations in hemostasis, leading to complex coagulation abnormalities [2]. Patients with cirrhosis frequently develop coagulopathy due to thrombocytopenia, decreased synthesis of coagulation factors, and alterations in their fibrinolytic pathways [3,4]. These combined disturbances contribute to the development of a fragile hemostatic balance, increasing the risk of both bleeding and thrombotic complications [5]. Although patients with cirrhosis have traditionally been perceived as having an increased bleeding tendency, studies published during the last 15 years have challenged this notion by highlighting the presence of a rebalanced yet unstable hemostatic system in these patients [6,7,8].

Standard coagulation (SC) blood tests using prothrombin time (PT), international normalized ratio (INR), activated partial thromboplastin time (aPTT), or serum fibrinogen have often demonstrated a hypocoagulable state in patients with cirrhosis and have traditionally helped medical practitioners decide whether to perform blood transfusions before procedures with a different bleeding risk [9]. However, these tests are limited, assessing only a small part of the coagulation cascade without counterbalancing the effects of anticoagulant changes, as they do not consider the influence of proteins C, S, and antithrombin III [8]. Consequently, relying on these tests can lead to unnecessary and potentially harmful transfusions without a corresponding reduction in the bleeding risk [10].

Rotational thromboelastometry (ROTEM) is a viscoelastic assay that evaluates the kinetics of blood clot formation and lysis in real time, evaluating clot initiation, propagation, maximum strength, and fibrinolysis [11]. In contrast with SC assays, thromboelastometry comprehensively assesses hemostatic function by capturing the dynamic interactions between the cellular elements and plasma components of blood, thereby offering a more physiologically relevant evaluation of the coagulation status [9,12]. Its use has been associated with improved transfusion practices and reduced blood product utilization in various surgical settings, including liver transplantation and cardiac surgery [13,14]. Currently, clinical guidelines do not recommend the use of routine coagulation tests in determining the indication for blood product transfusion before invasive procedures with a high bleeding risk; however, they also do not give firm recommendations regarding the use of ROTEM/thromboelastography (TEG) because few studies, with a heterogeneous population and different transfusion policies, support this [15,16,17]. Moreover, these recommendations are mostly sustained by expert opinion, as the currently available data are very limited [18].

The incidence of bleeding secondary to invasive endoscopic procedures in cirrhotic patients varies between 2% and 23%, depending on the type of intervention, the stage of liver cirrhosis, and the presence of predisposing factors, such as sepsis, AKI, or ACLF grade 3 [18,19,20,21,22]. The European guideline considers ERCP, endoscopic polypectomy, EUS with FNA/FNB, biliary dilatation, and variceal ligation as having a high risk of post-procedural bleeding, defining high risk as an incidence of bleeding complications above 1.5% [15].

Limited evidence exists to guide transfusion practices in the context of endoscopic procedures, which are common procedures but potentially with a high bleeding risk in decompensated cirrhotic patients. In this context, these procedures are often preceded by the prophylactic administration of blood components in response to abnormal PT or INR values, even though the relationship between these laboratory abnormalities and the actual bleeding risk remains uncertain [13]. Thromboelastometry-guided transfusion may offer a safer, more targeted approach that reduces the need for unnecessary transfusions and minimizes complications [23,24,25].

Several studies have evaluated TEG to guide blood transfusions before procedures in patients with cirrhosis, most showing a significant decrease in the need for transfusions [23,24]. Nevertheless, the data regarding the use of ROTEM-guided transfusion strategies in decompensated LC are scant [25].

This study aimed to evaluate the differences between SC- and ROTEM-based blood product transfusion algorithms in patients with decompensated LC undergoing high-bleeding-risk endoscopic procedures. We hypothesized that thromboelastometry-guided blood transfusion would result in fewer bleeding events and a lower transfusion burden, thereby improving patient safety and resource utilization.

## 2. Materials and Methods

This study was designed as a prospective cohort study and was conducted at a tertiary care university hospital between December 2024 and March 2025. The patients were followed up for 30 days after the endoscopic procedure. The protocol was approved by the Institutional Review Board (number: 491/4 November 2024), and written informed consent was obtained from all the participants.

### 2.1. Patients

In this study, we included all consecutive adult patients (>18 years of age) with a confirmed diagnosis of decompensated liver cirrhosis, scheduled for elective high-bleeding-risk endoscopic procedures with INR > 1.5 and/or platelets < 50 × 10^9^/L and/or fibrinogen < 100 mg/dL.

The exclusion criteria included active extrahepatic malignancies or hepatocellular carcinoma beyond the Milan criteria, a platelet count of <20 × 10^9^/L, acute-on-chronic liver failure, sepsis, known inherited bleeding disorders, ongoing anticoagulant or antiaggregant therapy, the receipt of a blood product transfusion in the last 7 days, stage 4 or 5 chronic kidney disease, and refusal to participate.

This study recorded baseline demographic data, the liver disease etiology, and Child–Pugh (CP) and Model for End-Stage Liver Disease (MELD) scores. A cirrhosis diagnosis was established using a combination of clinical, biochemical, and imaging findings, with histological confirmation obtained when available. Decompensated LC was defined as the presence of at least one of the following: ascites, hepatic encephalopathy, and/or variceal bleeding [26]. Mild–moderate ascites was defined as the presence of intraperitoneal fluid detected using ultrasound or physical examination (shifting dullness and fluid wave). Patients with tense abdomens due to a large volume of fluid were considered to have severe ascites [26]. Hepatic encephalopathy (HE) was classified using the West Haven scale [27]. Bleeding complications were categorized as major or minor per the International Society on Thrombosis and Haemostasis’s recommendations [28].

All the transfusions were performed before the endoscopic procedures, and the coagulation parameters were not rechecked. The patients were followed up for 30 days after the procedure in the outpatient clinic or via telephone.

### 2.2. Study Groups

ROTEM group: Transfusion decisions were made according to a predefined ROTEM algorithm. Cryoprecipitate was given if the FIBTEM A5 was less than 9 mm; platelet transfusion was provided if the EXTEM clot amplitude (A10) was less than 40 mm; and fresh frozen plasma (FFP) was considered if the clotting time (CT) was more than 80 s in the EXTEM test. The thromboelastometry-guided transfusion protocol was adapted from that of Görlinger et al. [29], utilizing the EXTEM and FIBTEM assays provided by ROTEM^®^ (ROTEM, TEM, Munich, Germany). Transfusion was considered unnecessary when the CT-EXTEM was ≤80 s and the EXTEM amplitude at 10 min (A10) was ≥40 mm. In cases where the CT-EXTEM exceeded 80 s, fresh frozen plasma (FFP) was administered at a dose of 10 mL per kilogram of body weight. If the A5 value in the FIBTEM assay was ≥9 mm, indicative of sufficient fibrinogen function, platelet transfusion was administered at a dose of 1 unit per 10 kg of body weight, up to a maximum of 10 units. Conversely, if the A5-FIBTEM was <9 mm, reflecting fibrinogen deficiency, cryoprecipitate was administered at the same dosage. Management of patients in both the ROTEM-guided and standard care groups was identical, except for the approach used to determine the prophylactic administration of blood components.

In the SC group, transfusion decisions were based on the standard levels: if the platelet count was less than 50 × 10^9^/L, the INR was over 1.5, or the fibrinogen was below 100 mg/dL, then a platelet transfusion, FFP, or cryoprecipitate was given, respectively. If the INR exceeded 1.5, fresh frozen plasma (FFP) was administered at a dose of 10 mL per kilogram of body weight. If the platelet count was <50 × 10^9^/L, a platelet transfusion was administered at a dose of 1 unit per 10 kg of body weight, up to a maximum of 10 units. Similarly, if the serum fibrinogen was <100 mg/dL, cryoprecipitate was administered at the same dose and with the same maximum limit.

The blood was collected in a Vacutainer with 3.2% sodium citrate via a butterfly needle. The samples were analyzed within an hour after collection.

### 2.3. Endoscopic Procedures and Bleeding Management

All endoscopic procedures were performed by six expert endoscopists.

We documented any episodes of postoperative bleeding. Immediate bleeding after an endoscopic procedure was defined as any bleeding that occurred during the procedure itself or immediately upon completion of the endoscopic intervention, before the patient left the endoscopy unit. The rest of the bleeding complications were considered delayed bleeding complications.

Endoscopic hemostasis included hemoclipping, electrocauterization, and/or epinephrine injection as required.

### 2.4. Outcome Measures

- Primary Outcomes: The total amount of blood products given and the number of adverse events caused by transfusions.
- Secondary Outcomes: The incidence of immediate and delayed bleeding complications, length of hospital stay, and mortality within 30 days.

### 2.5. Rotational Thromboelastometry Method

The ROTEM test was conducted per the manufacturer’s guidelines following the collection of a venous blood sample from the antecubital vein. The test was conducted using a native blood sample with a fully automated ROTEM sigma machine (ROTEM, TEM, Munich, Germany) before the elective procedure. The ROTEM machine processed the sample for 30 min, determining and automatically recording various parameters. The ROTEM manufacturer was not involved in this study. No equipment, technical support, funding, or any form of financial or non-financial support was provided by the company.

#### ROTEM Parameters

The duration between sample recalcification and activation of clot formation is known as the clotting time, and it is longer in patients undergoing oral anticoagulation or heparin therapy or who have coagulation deficits. The kinetics of clot formation are described by the alpha angle and clot formation time, while the platelet count and fibrinogen levels influence the maximum clot stiffness. The current study evaluated the results from two tests: EXTEM produces a result similar to the INR, as it involves adding a tissue factor to induce coagulation, and FIBTEM shows the highest clot strength amplitude after 5 min (A5), accurately showing how much fibrinogen is present because it starts by adding a tissue factor and uses a platelet blocker (cytochalasin D).

### 2.6. Statistical Analysis

A computer-generated sequence was used as an allocation tool to minimize selection bias and ensure a balanced distribution between groups. However, this process was not part of a formal randomized controlled trial design. This study did not include allocation concealment, blinding, or other methodological features required for randomized interventional studies. Therefore, this study was an observational prospective cohort study with bias-reduced allocation. A total of at least 56 participants (28 in each arm) was needed, assuming a minimum 30% difference in transfusion requirement between the study groups, with a 5% alpha error and 80% power, and a 10% dropout.

The data were analyzed with SPSS software version 20.0 (SPSS Inc., Chicago, IL, USA). We expressed continuous variables as means, standard deviations (SDs), or medians (IQRs) and compared them using Student’s *t*-test or the Mann–Whitney U test, as appropriate. Categorical variables were analyzed using the chi-square test or Fisher’s exact test. In this study, *p* < 0.05 was set as the significance level.

## 3. Results

**Figure 1:**
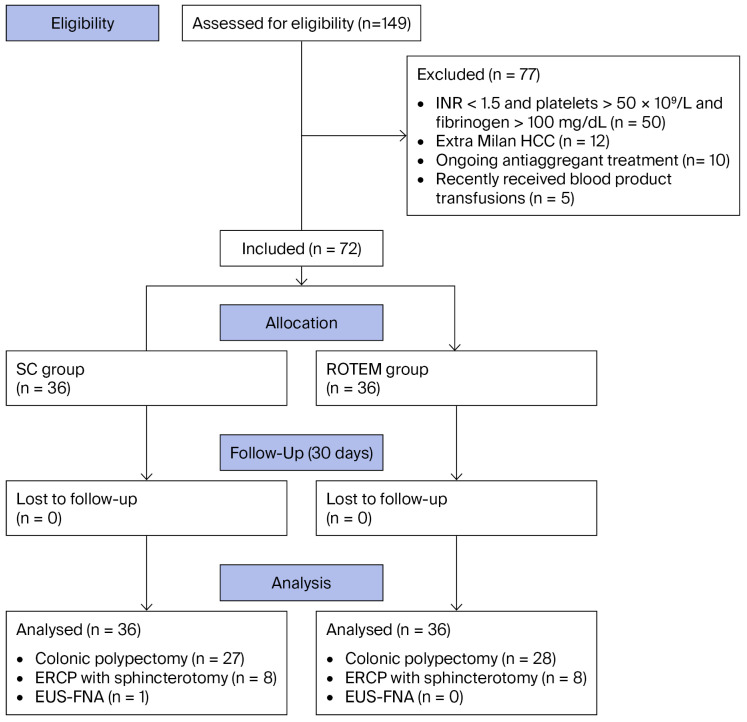
Flow diagram of this study.

During this study, we screened 149 patients. Seventy-seven patients were excluded (fifty patients had INR < 1.5, platelets > 50 × 10^9^/L, and fibrinogen > 100 mg/dL; twelve patients presented with extra-Milan HCC; ten patients were undergoing antiaggregant treatment; and five patients had recently received blood product transfusions) (Figure 1). Seventy-two patients with decompensated liver cirrhosis undergoing high-bleeding-risk endoscopic procedures were included in this study, with 36 patients in each group.

In the entire patient cohort, the mean age was 53.6 ± 0.7 years; most of them were male (63.9%), with predominant alcoholic liver cirrhosis (51.4%). Most patients (64 patients (88.8%)) were on the liver transplantation waiting list.

There were no significant differences between the two groups regarding their age (*p* = 0.882), sex (*p* = 0.624), causes of liver cirrhosis (*p* = 0.234), or liver disease severity, as shown by the Child–Pugh (*p* = 0.704) and MELD scores (*p* = 0.793). Most of the patients had mild–moderate ascites (75.0%) and hepatic encephalopathy (52.8%). None of the patients included in this study had upper or lower digestive hemorrhage, hepato-renal syndrome, or infectious complications of liver cirrhosis.

Most patients had colonic polypectomy (76.4%), 16 patients had endoscopic retrograde cholangiopancreatography (ERCP) with sphincterotomy (22.2%), and only one patient had endoscopic ultrasound with fine-needle aspiration (EUS-FNA) (1.4%), with no statistically significant differences between the two study groups (*p* = 0.601) (Table 1).

The mean number of polyps was 1.09 ± 0.34, with no differences in Child–Pugh class (CP-A 1.09 ± 0.38 vs. CP-B 1.09 ± 0.29; *p* = 0.948) or the study group (ROTEM group 1.07 ± 0.38 vs. 1.1 ± 0.32; *p* = 0.677). Most patients had polyps between 1 and 2 cm (48 patients (87.3%)), situated on the left side of the colon (72.9%). Polypectomies were performed for polyps of more than 2 cm in only seven patients (12.7%), all the polyps being situated in the descending and transverse parts of the colon. Epinephrine was injected into the polyp’s base before hot snare cutting in all endoscopic polypectomies.

The median INR was elevated in both groups (1.69 vs. 1.80; *p* = 0.301), consistent with cirrhotic coagulopathy. The median platelet count was 54.5 × 10^9^/L in the ROTEM group compared with 55.5 × 10^9^/L in the SC group (*p* = 0.839), and the fibrinogen levels averaged 172.2 ± 35.6 mg/dL in the ROTEM group and 155.1 ± 43.2 mg/dL in the SC group (*p* = 0.073).

In the thromboelastometry group, the ROTEM analysis revealed a mean EXTEM CT of 70.8 ± 1.1 s, a median EXTEM A10 of 42 mm, and a median FIBTEM A5 of 12 mm.

### 3.1. Preemptive Blood Product Transfusion

The patients in the SC group received significantly more blood products compared with those in the ROTEM group (*p* = 0.0001). Twenty-seven (37.5%) patients received FFP, of whom 25 (3.8%) were from the ROTEM group and eight (30.8%) were from the SC group (*p* < 0.0001) (Table 2). A total of 21 (29.2%) patients received platelets, of whom five (13.9%) were in the ROTEM group, and 16 (44.4%) were in the SC group (*p* = 0.004). In total, eleven (15.3%) patients received cryoprecipitate. The patients in the ROTEM group received cryoprecipitate more frequently than those in the SC group (25.0% vs. 5.6%; *p* = 0.022). No thrombotic complications were recorded.

Overall, seven (9.7%) patients required both FFP and platelet transfusion, all of them from the SC group. None of the patients included in this study received tranexamic acid.

### 3.2. Primary and Secondary Outcomes

In the SC group, there were three (8.3%) transfusion-related adverse events: one transfusion-related acute lung injury (TRALI) in a cirrhotic patient who received both FFP and platelet transfusion, and two allergic reactions, which were treated with corticotherapy. One (2.8%) transfusion-related adverse event occurred in the ROTEM group: an allergic reaction after plasma transfusion. No thrombotic events were recorded in either study group.

Twelve patients (16.7%) developed bleeding complications, including four in the ROTEM group and eight patients in the SC group (*p* = 0.206). Among patients who experienced bleeding, all patients in the SC group and three out of four in the ROTEM group had received prophylactic blood product transfusion. The bleeding events were considered minor, and no blood product transfusions were needed.

There were no significant differences in the frequency of immediate or delayed bleeding between the two study groups (*p* = 0.217 and *p* = 0.584, respectively). All bleeding was stopped using hemoclipping and/or electrocauterization. Moreover, there were no differences in the prophylactic transfusion of blood products between patients with and without bleeding complications (*p* = 0.075).

The length of hospital stay was similar in both groups, with a median of 7 days (*p* = 0.618). During the 30-day follow-up, five (6.9%) patients died, comprising three (8.3%) in the SC group and two (5.6%) in the ROTEM group (*p* = 0.643). All the deaths were caused by complications related to liver cirrhosis, including variceal bleeding in two patients and sepsis with acute-on-chronic liver failure in three patients. All the patients who died within 30 days had MELD scores of more than 20 at baseline.

## 4. Discussion

In recent decades, cirrhosis has no longer been considered a disease characterized by hypocoagulability and a high bleeding risk [9]. Thus, it has been demonstrated that invasive procedures have a bleeding risk similar to that of the general population, and the correction of coagulation disorders identified using routine tests is not always indicated [30]. However, there are few data on validated methods that adequately assess both the bleeding risk and the indication for transfusions, especially in patients with decompensated cirrhosis. Moreover, most of the current recommendations are based on expert opinion, and most of the data are extracted from retrospective cohort studies, including a wide range of invasive procedures [21,30]. All these data highlight the need to focus on specific high-bleeding-risk procedures in patients with liver cirrhosis, especially since post-procedural bleeding is associated with an increased risk of 28-day mortality [30], and most cirrhotic patients tend to receive unnecessary prophylactic blood product transfusions before invasive procedures, increasing the risk of alloimmunization and graft rejection after liver transplantation.

This study evaluated differences between a ROTEM-guided and a conventional laboratory-based blood product transfusion strategy in patients with decompensated liver cirrhosis undergoing high-bleeding-risk endoscopic procedures. We extended the ROTEM algorithm used for liver transplantation to explore its applicability for high-bleeding-risk endoscopic procedures in patients with decompensated liver cirrhosis on the liver transplantation list [31]. Our findings suggest that the use of a ROTEM-based transfusion algorithm was associated with lower blood product transfusion rates compared to a standard threshold-based approach. However, these results should be interpreted cautiously, given the methodological limitations of the study and the exploratory nature of the analysis. No significant differences were observed between groups regarding baseline characteristics, including liver disease severity (Child–Pugh and MELD scores), etiology, and the type of endoscopic procedures, which are known to influence bleeding risk. This comparability supports that the observed differences in outcomes are unlikely to be confounded by these factors. Our data show that a ROTEM-based strategy decreased the need for blood product transfusion. Moreover, our study demonstrated that the ROTEM group received cryoprecipitate more frequently than the SC group, as well as a lower volume of blood products. Furthermore, the lower transfusion rate observed in the ROTEM group may partly reflect differences in transfusion triggers between the two strategies, rather than a purely clinical effect. As transfusion decisions in the ROTEM group were guided by viscoelastic parameters, while those in the standard group were based on conventional laboratory thresholds, this methodological difference may introduce a degree of assessment bias. Therefore, the observed reduction in transfusion requirements should be interpreted with caution, as this could be an effect of the transfusion algorithm itself rather than evidence of diagnostic superiority. Importantly, the SC group was managed using predefined laboratory thresholds (INR, platelet count, and fibrinogen), which may be considered more intervention-prone than current guideline-based practice. As a result, the likelihood of transfusion in the SC group may have been increased by design. Therefore, the observed difference in transfusion frequency between groups should not be interpreted primarily as evidence that ROTEM more accurately identifies patients who require transfusion. Instead, it reflects a comparison between two distinct transfusion strategies. Three retrospective and one prospective study have evaluated the bleeding risk following invasive procedures in patients with liver cirrhosis [20,21,30,32]. The bleeding risk in patients diagnosed with decompensated liver cirrhosis who underwent invasive endoscopic procedures ranged from 2.3% to 8.3%. Prophylactic administration of blood products based on standard coagulation tests did not influence the bleeding rate, confirming that these tests cannot be used to guide blood product transfusion in decompensated liver cirrhosis [20,21,32]. Giannini et al. evaluated patients with decompensated CH, with a mean MELD score of 22, and demonstrated a 6% bleeding risk following endoscopic procedures [20]. Furthermore, in a prospective, multicenter cohort study, Intagliata et al. demonstrated that patients diagnosed with decompensated LC undergoing endoscopic procedures carried a 14% bleeding risk. The risk ranged from 0.7% for endoscopic ultrasound with biopsy to 15.4% for colonoscopy with polypectomy. A MELD score of more than 25, BMI > 40, and high-bleeding-risk procedures are associated with an increased bleeding risk after non-surgical invasive procedures [30]. Moreover, bleeding was not influenced by preprocedural blood product prophylaxis based on conventional coagulation tests; these data were confirmed in our study.

Despite a longstanding reliance on traditional coagulation tests, such as those determining the INR and platelet counts, these measures have well-documented limitations in cirrhotic patients [6,9,15]. SOC coagulation tests do not reflect the complex rebalanced hemostatic state typical of advanced liver disease, often leading to overestimation of the bleeding risk and subsequent overtreatment [3,12,17,23,24,25]. Our data support this: the patients managed using standard coagulation tests received significantly more blood products than those in the ROTEM-guided group, with no statistically significant reduction in bleeding complications. The data on this topic are still scant. Some studies have demonstrated that TEG-based transfusion strategies are safe and reduce the need for preprocedural blood product transfusion in cirrhotic patients who need to undergo procedures with a high bleeding risk [23,24]. Regarding central venous catheter insertion in liver cirrhosis patients, Rocha et al. [33] also demonstrated a decrease in blood product transfusion in an ROTEM-guided cohort.

Both ROTEM and TEG are viscoelastic tests assessing whole-blood clot dynamics. The fundamental difference lies in mechanics: TEG rotates the cup while ROTEM rotates the pin. ROTEM offers pathway-specific assays and greater automation than TEG. Clinically, both guide transfusion and bleeding management, though ROTEM provides more standardized, reagent-specific information and is easier to use at the bedside. In our study, 58.8% of the patients in the ROTEM arm did not need any transfusions compared with the SC group, in which all the patients received blood product transfusions. Our results are similar to other published data using TEG in cirrhotic patients for different invasive procedures [14,23,24,25,34,35]. In two randomized studies using TEG as a viscoelastic test, it was demonstrated that in cirrhotic patients undergoing procedures with a low–intermediate risk of peri-procedural bleeding, the TEG group received significantly fewer blood products than the SC group [23,24].

Studies using ROTEM also demonstrated a decreased number of transfusions; however, these results were obtained in a population of cirrhotic patients with high MELD scores, most of whom were decompensated, a population similar to that included in our study [25,34,35]. Nevertheless, these studies were heterogeneous in terms of the type and degree of risk of the invasive procedures evaluated, unlike the population included in our study.

Our study included three high-bleeding-risk procedures: ERCP with sphincterotomy, EUS with FNA, and endoscopic polypectomy, the last being the most frequent (76.4%). In the general population, post-colonic polypectomy bleeding ranges between 0.2 and 6.1% [36,37], and polyps larger than 1 cm in the right colon, laterally spreading tumors, multiple polyps, and underlying comorbidities have been identified as risk factors for post-procedural bleeding.

In a retrospective cohort, Soh et al. demonstrated that 21.9% of patients with decompensated liver cirrhosis developed bleeding complications after colonoscopic polypectomy, and the risk of late bleeding was 20 times higher than in the general population [19]. Our results reported lower rates of immediate (15.3%) and delayed bleeding complications (2.8%) in a similar study population with regard to LC severity and the complexity of endoscopic procedures, emphasizing the importance of correct indication of preemptive blood product transfusion.

Although current guidance/guidelines from major societies do not recommend conventional coagulation tests (INR, platelets, and fibrinogen) to guide preemptive blood product transfusion because these parameters do not reliably predict bleeding [15,38,39,40,41], they also do not clearly recommend ROTEM/TEG to identify patients who can safely avoid transfusion, especially in decompensated cirrhotic patients [15,41], in whom case-by-case decisions are still indicated. Our findings align with the increasing recognition that SC tests incompletely capture the rebalanced hemostasis of cirrhosis, whereas ROTEM could identify patients who can avoid prophylactic products.

There were no statistically significant differences in the SC test coagulation parameters between the two study groups, as per the previous literature showing that viscoelastic testing, such as that using ROTEM, could identify cirrhotic patients who are hemostatically competent despite abnormal standard lab results [11,13]. This focused approach could help prevent unnecessary blood transfusions and the associated risks, such as having excessive fluid in the body, lung injuries from the transfusion, and immune reactions.

This study also highlights the feasibility of applying ROTEM-guided algorithms in outpatient decompensated LC requiring colonoscopy with polypectomy or therapeutic ERCP with sphincterotomy, which are particularly relevant in patients awaiting liver transplantation, in whom colonic cancer screening and prevention and treatment of choledochal lithiasis are equally important. Although the baseline fibrinogen levels and platelet counts were relatively similar between the groups, the thromboelastometry group received fewer FFP and platelet transfusions. These findings suggest that ROTEM parameters such as FIBTEM A5 and EXTEM A10 could offer better guidance than absolute counts, aligning with studies advocating for the use of functional assays in liver disease patients [11,25,29,42]. However, it is essential to distinguish between the operational effect of a transfusion algorithm and the diagnostic performance of the underlying test. While ROTEM-guided management modifies transfusion practice, our study does not establish that ROTEM more accurately identifies true transfusion need or bleeding risk. Moreover, the ROTEM-guided approach relies on viscoelastic parameters that may be more permissive and individualized.

Our results demonstrated a tendency toward more frequent use of cryoprecipitate transfusion in the ROTEM group. These data have also been confirmed by other studies [23,25], which raised concerns regarding thrombotic complications of using cryoprecipitate [43], as it contains fibrinogen as well as factors VIII and XIII and the von Willebrand factor. No thrombotic events were reported in our study, confirming recently published data on using ROTEM-based transfusion strategies in patients with liver cirrhosis [25].

One patient developed TRALI in our SC group, and three patients developed allergic reactions in the SC group, while none did in the ROTEM group. The frequency in the whole cohort was higher than the previously reported rates of 2–2.5% [25,44]. The higher allergic reaction rates in our group could be partially explained by the inclusion of patients with more severe cirrhosis and previous exposure to blood products, who are prone to developing antiplatelet antibodies and immune reactions.

The 30-day mortality rate was the same in both groups, even though the total volume of blood products transfused was reduced in the ROTEM group. Our results align with other studies, where the mortality was not influenced by the blood product transfusion strategy or the length of hospital stay [25,42]. However, we included patients on the liver transplantation waiting list, in whom it is vital to reduce the amount of blood products transfused before liver transplantation to decrease the rejection risk after the transplant procedure. Although bleeding events were numerically fewer in the ROTEM group, this difference was not statistically significant, and the study was not powered to detect differences in bleeding complications, mortality, or other clinically relevant outcomes.

A major strength of our study is that, to our knowledge, it is the first to evaluate ROTEM-based blood product transfusion in a homogeneous cirrhotic population needing high-bleeding-risk endoscopic procedures, with a prospective follow-up of 30 days and no dropouts.

Our study also had limitations. It was conducted at a single center and did not include randomization, allocation concealment, or blinding, which may introduce bias. Our study population consisted exclusively of patients with decompensated liver cirrhosis, a subgroup characterized by a complex and unstable hemostatic balance. In this setting, current guidelines do not provide clear recommendations regarding the use of either conventional coagulation tests or viscoelastic assays, and instead suggest that transfusion decisions should be individualized on a case-by-case basis. Therefore, our findings should be interpreted in the context of this clinical uncertainty, and further studies are needed to compare ROTEM-guided strategies with more restrictive, guidelines-based transfusion approaches. The standard coagulation arm was managed using fixed laboratory thresholds that may not reflect contemporary guideline-based practice because there are no clear recommendations in regard to coagulopathy management in patients with decompensated liver cirrhosis. This may have increased transfusion exposure and amplified the observed difference between groups. The sample size, while sufficient to detect trends, may have been insufficient for identifying small but significant differences in the bleeding outcomes. Moreover, there are no clear baseline values for ROTEM parameters in cirrhotic patients. Although our study observed fewer bleeding events in the ROTEM group, this difference did not reach statistical significance. Therefore, our results do not support a definitive conclusion regarding the reduction in bleeding complications. Another important limitation is that this study was likely underpowered to detect differences in bleeding outcomes due to the relatively small sample size and low event rate; therefore, these results should be interpreted with caution, which limits generalizability. Furthermore, the comparison between the ROTEM group and the standard-of-care group for FFP transfusion is limited, since the control group was managed according to transfusion practices that differ from current guideline recommendations. While ROTEM-guided management was associated with reduced transfusion requirements, this finding should be interpreted cautiously, as differences in the transfusion criteria between groups may have contributed to this effect. In addition, this was a single-center study, which may limit the generalizability of the findings. More studies at different centers are needed to confirm these results and determine how cost-effective ROTEM-guided protocols are in various medical settings. Considering these limitations, the findings should be interpreted as exploratory and hypothesis-generating rather than definitive.

Given the absence of manufacturer involvement in the study, data collection, analysis, or interpretation, we aimed to ensure an independent evaluation. Nevertheless, interpretation of the results must remain neutral and proportionate to the strength of the evidence. The findings should not be interpreted as supporting routine clinical adoption of ROTEM-guided blood product transfusion strategies based on the present data alone.

Overall, the findings of this study should be considered preliminary and hypothesis-generating. The single-center design, relatively small sample size, lack of blinding, limited power for clinical outcomes, and comparator-related constraints restrict the strength and generalizability of the conclusions. Future large-scale, multicenter, randomized studies are required to determine whether ROTEM-guided blood product transfusion improves clinical outcomes or more accurately identifies decompensated cirrhotic patients who benefit from transfusion before high-risk-of-bleeding endoscopic procedures.

## 5. Conclusions

A ROTEM-based blood product transfusion algorithm was associated with reduced blood product utilization compared to a conventional laboratory threshold-based approach in patients with decompensated liver cirrhosis undergoing high-risk-of-bleeding endoscopic procedures. However, this finding should be interpreted with caution, as it likely reflects differences in transfusion strategies, including a potentially more intervention-prone comparator, rather than a demonstrated intrinsic advantage of ROTEM. No significant differences were observed in bleeding complications, mortality, or other clinically relevant outcomes, and no conclusions can be drawn regarding diagnostic superiority. Therefore, these results should be considered preliminary and hypothesis-generating. Further adequately powered, multicenter randomized studies are required to determine the clinical value of ROTEM-guided blood product transfusion management in patients with decompensated liver cirrhosis undergoing high-risk-of-bleeding endoscopic procedures.

## Figures and Tables

**Table 1:** Baseline patient characteristics.

Parameter	All Patients *N* = 72	ROTEM Group *N* = 36	SC Group *N* = 36	*p*-Value
Age (years), mean ± SD	53.6 ± 0.7	53.5 ± 1.0	53.7 ± 1.1	0.882
Male gender, *n* (%)	46 (63.9)	24 (66.6)	22 (61.1)	0.624
Etiology, *n* (%)				0.234
Alcohol	37 (51.4)	16 (44.4)	21 (58.3)	
Viral	22 (30.6)	14 (38.9)	8 (22.2)	
Autoimmune	7 (9.7)	2 (5.6)	5 (13.9)	
MASH	6 (8.3)	4 (11.1)	2 (5.6)	
Ascites, *n* (%)				0.951
Mild	13 (18.1)	7 (19.4)	6 (16.7)	
Moderate	41 (56.9)	20 (55.6)	21 (58.3)	
Severe	18 (25.0)	9 (25.0)	9 (25.0)	
HE, *n* (%)	38 (52.8)	16 (44.4)	22 (61.1)	0.209
Hemoglobin (g/dL), median (IQR)	11.3 (2)	11.4 (2.15)	11.2 (2.3)	0.401
INR, median (IQR)	1.72 (0.3)	1.69 (0.24)	1.80 (0.78)	0.301
Platelets (×10^9^/L), median (IQR)	55.0 (36.0)	54.5 (33.0)	55.5 (35.8)	0.839
Fibrinogen (g/dL), mean ± SD	163.6 ± 40.4	172.2 ± 35.6	155.1 ± 43.2	0.073
Creatinine (mg/dL), mean ± SD	0.68 ± 0.16	0.7 ± 0.78	0.67 ± 0.54	0.500
Albumin (g/dL), median (IQR)	3.1 (0.4)	3.1 (0.58)	3.05 (0.4)	0.207
Child–Pugh class B/C, *n* (%)	39/33 (54.2/45.8)	19/17 (52.8/47.2)	20/16 (55.6/44.4)	0.813
Child–Pugh score, median (IQR)	9 (2)	9 (2)	9 (2)	0.704
MELD score, median (IQR)	18 (4)	18 (4)	18 (4)	0.793
Type of endoscopic procedure, *n* (%)				
Colonic polypectomy	55 (76.4)	28 (77.8)	27 (75.0)	0.601
ERCP with sphincterotomy	16 (22.2)	8 (22.2)	8 (22.2)	
EUS with FNA	1 (1.4)	0 (0)	1 (2.8)	

Abbreviations: ERCP—endoscopic retrograde cholangiopancreatography; EUS—endoscopic ultrasound; FNA—fine-needle aspiration; HE—hepatic encephalopathy; INR—international normalized ratio; MASH—metabolic-associated steatohepatitis; MELD—Model of End-Stage Liver Disease; ROTEM—rotational thromboelastometry; SD—standard deviation; SC—standard coagulation.

**Table 2:** Coagulation parameters and outcomes.

Parameter	All Patients *N* = 72	ROTEM Group *N* = 36	SC Group *N* = 36	*p*-Value
INR, median (IQR)	1.72 (0.3)	1.69 (0.24)	1.80 (0.78)	0.301
Platelets (×10^9^/L), median (IQR)	55.0 (36.0)	54.5 (33.0)	55.5 (35.8)	0.839
Fibrinogen (g/dL), mean ± SD	163.6 ± 40.4	172.2 ± 35.6	155.1 ± 43.2	0.073
EXTEM CT (s), mean ± SD	70.8 ± 1.1	70.8 ± 1.1	-	
EXTEM A10 (mm), median (IQR)	42.0 (6)	42.0 (6)	-	
FIBTEM A5 (mm), median (IQR)	12 (4)	12 (4)	-	
Preemptive FFP transfusion, *n* (%)	27 (37.5)	2 (5.6)	25 (69.4)	**<0.0001**
Preemptive platelet transfusion, *n* (%)	21 (29.2)	5 (13.9)	16 (44.4)	**0.004**
Preemptive cryoprecipitate transfusion, *n* (%)	11 (15.3)	9 (25.0)	2 (5.6)	**0.022**
Transfusion of two blood products, *n* (%)	7 (9.7)	0 (0)	7 (19.4)	**0.005**
Preemptive transfusion, *n* (%)	51 (70.8)	15 (41.2)	36 (100)	**<0.0001**
Bleeding complications, *n* (%)	12 (16.7)	4 (11.1)	8 (22.2)	0.206
Immediate bleeding complications, *n* (%)	11 (15.3)	4 (11.1)	7 (19.4)	0.217
Delayed bleeding complications, *n* (%)	2 (2.8)	1 (2.8)	1 (2.8)	0.584
Transfusion-related adverse events, *n* (%)	4 (5.6)	1 (2.8)	3 (8.3)	0.713
Length of hospital stay (days), median (IQR)	7 (4)	7 (3)	7 (4)	0.618
Death at 30 days, *n* (%)	5 (6.9)	2 (5.6)	3 (8.3)	0.643

Abbreviations: INR—international normalized ratio; ROTEM—rotational thromboelastometry; SD—standard deviation; SC—standard coagulation. Bold values are considered statistically significant.

## Data Availability

The data presented in this study are available upon request from the corresponding author. The data are not publicly available because they are the property of the Institute of Gastroenterology and Hepatology, Iasi, Romania.

## Abbreviations

The following abbreviations were used in this manuscript.

SC	Standard coagulation
PT	Prothrombin time
INR	International Normalized Ratio
aPTT	Activated partial thromboplastin time
ROTEM	Rotational thromboelastometry
TEG	Thromboelastography
FFP	Fresh frozen plasma
CT	Clotting time
MELD	Model for End-Stage Liver Disease
SD	Standard deviation
IQR	Interquartile range
HE	Hepatic encephalopathy
ERCP	Endoscopic retrograde cholangiopancreatography
EUS	Ultrasound endoscopy
FNA	Fine-needle aspiration
LC	Liver cirrhosis
TRALI	Transfusion-related acute lung injury
MASH	Metabolic-associated steatohepatitis
